# Effects of two alveolar recruitment maneuvers in an “open-lung” approach during laparoscopy in dogs

**DOI:** 10.3389/fvets.2022.904673

**Published:** 2022-08-18

**Authors:** Caterina Di Bella, Caterina Vicenti, Joaquin Araos, Luca Lacitignola, Laura Fracassi, Marzia Stabile, Salvatore Grasso, Alberto Crovace, Francesco Staffieri

**Affiliations:** ^1^School of Bioscience and Veterinary Medicine, University of Camerino, Camerino, Italy; ^2^Section of Veterinary Clinics and Animal Production, Department of Emergency and Organ Transplantations, University of Bari, Bari, Italy; ^3^Department of Clinical Sciences, College of Veterinary Medicine, Cornell University, Ithaca, NY, United States; ^4^Section of Anesthesia and Intensive Care, Department of Emergency and Organ Transplantations, University of Bari, Bari, Italy

**Keywords:** laparoscopy, atelectasis, alveolar recruitment, oxygenation, dog

## Abstract

**Objectives:**

The aim of this study was to compare the effects of a sustained inflation alveolar recruiting maneuver (ARM) followed by 5 cmH_2_O of PEEP and a stepwise ARM, in dogs undergoing laparoscopic surgery.

**Materials and methods:**

Twenty adult dogs were enrolled in this prospective randomized clinical study. Dogs were premedicated with methadone intramuscularly (IM); anesthesia was induced with propofol intravenously (IV) and maintained with inhaled isoflurane in pure oxygen. The baseline ventilatory setting (BVS) was as follows: tidal volume of 15 mL/kg, inspiratory pause of 25%, inspiratory to expiratory ratio of 1:2, and the respiratory rate to maintain the end-tidal carbon dioxide between 45 and 55 mmHg. 10 min after pneumoperitoneum, randomly, 10 dogs underwent sustained inflation ARM followed by 5 cmH_2_O of PEEP (ARMi), while 10 dogs underwent a stepwise recruitment maneuver followed by the setting of the “best PEEP” (ARMc). Gas exchange, respiratory system mechanics, and hemodynamic were evaluated before the pneumoperitoneum induction (BASE), 10 min after the pneumoperitoneum (PP), 10 min after the recruitment (ARM), and 10 min after the pneumoperitoneum resolution (PostPP). Statistical analysis was performed with the ANOVA test (*p* < 0.05).

**Results:**

Static compliance decreased in both groups at PP (ARMc = 1.35 ± 0.21; ARMi = 1.16 ± 0.26 mL/cmH_2_O/kg) compared to BASE (ARMc = 1.78 ± 0.60; ARMi = 1.66 ± 0.66 mL/cmH_2_O/kg) and at ARM (ARMc = 1.71 ± 0.41; ARMi = 1.44 ± 0.84 mL/cmH_2_O/kg) and PostPP (ARMc = 1.75 ± 0.45; ARMi = 1.89 ± 0.59 mL/cmH_2_O/kg), and it was higher compared to PP and similar to BASE. The PaO_2_/FiO_2_, in both groups, was higher at ARM (ARMc = 455.11 ± 85.90; ARMi = 505.40 ± 31.70) and PostPP (ARMc = 521.30 ± 66.20; ARMi = 450.90 ± 70.60) compared to PP (ARMc = 369.53 ± 49.31; ARMi = 394.32 ± 37.72).

**Conclusion and clinical relevance:**

The two ARMs improve lung function in dogs undergoing laparoscopic surgery similarly. Application of PEEP at the end of the ARMs prolonged the effects of the open-lung strategy.

## Introduction

Laparoscopic surgery has become a common approach for abdominal surgical procedures in dogs (1–3). The type of surgery, the level of intra-abdominal pressure (IAP) to generate pneumoperitoneum (PP), and its duration may compromise the cardiovascular and respiratory systems (4, 5). Changes associated with PP include alterations in arterial blood pressure (ABP), reductions in cardiac output (CO) and respiratory system compliance, and lung compression due to the cranial displacement of the diaphragmatic dome (6–9). Moreover, the absorption of carbon dioxide (CO_2_) during PP can result in hypercarbia and respiratory acidosis (10, 11). The use of mechanical ventilation is therefore essential during laparoscopic procedures (12, 13).

General anesthesia with muscle paralysis, tracheal intubation, and intermittent positive pressure ventilation (IPPV) is often used to guarantee the target minute ventilation during laparoscopy (14, 15). Moreover, several strategies have been tested aimed at improving respiratory function and oxygenation, such as the application of positive end-expiratory pressure (PEEP) (16), which increases the functional residual capacity (FRC) and stabilizes the alveolar units (17, 18). The open-lung concept (OLC) refers to a strategy aimed at opening non-aerated or poorly aerated alveolar units by increasing the transpulmonary pressure (PL) of the lungs. This increased PL overcomes the closing pressure of collapsed alveoli, thus causing their re-expansion (18–20). The goal is to minimize cyclic forces associated with repetitive alveolar collapse and re-opening, which has been associated with suboptimal gas exchange and generation of lung inflammation (21–23).

Currently, two main types of alveolar recruitment maneuvers (ARM) have been described to improve lung mechanics and arterial oxygenation in dogs: the sustained inflation (or vital capacity) ARM (24–26) and the stepwise ARM (22, 27). The application of PEEP at the end of the ARM is critical to stabilize the reopened alveolar units and to keep them open. With the sustained inflation ARM, the PEEP level following the ARM is fixed and established by the operator (24–31). With the stepwise approach, the level of PEEP is individualized based on the specific respiratory mechanical properties of patient (best PEEP) (22, 23, 30, 32). In the last years, several studies have shown that both types of ARMs are effective in improving respiratory mechanics and gas exchange in the intraoperative period (22, 23, 31). However, to the authors' knowledge, the efficacy of the two maneuvers has never been compared in dogs undergoing laparoscopic surgery.

The aim of this study was to evaluate the effects of a sustained inflation (ARMi) maneuver followed by 5 cmH_2_O of PEEP compared to a stepwise (ARMc), in which the best PEEP was identified as the minimum PEEP level resulting in the highest static compliance. We hypothesized that ARMc, due to case-specific optimization of the PEEP level, would be more effective in improving respiratory system compliance and gas exchange, limiting the cardiovascular side effects, in anesthetized dogs, undergoing laparoscopic surgery. To test our hypothesis, respiratory system mechanics, hemodynamic, and gas exchange parameters were studied and compared at predetermined times of the study in dogs undergoing elective laparoscopic gastropexy.

## Materials and methods

This prospective, randomized clinical study was approved by the Ethical Committee for Clinical Study in Animal Patients of the Department Emergency and Organ Transplantation of the University of Bari (No. 03/2016). The manuscript is reported following the CONSORT Statement 2010 for reporting randomized controlled trials (33).

### Patients

Adult, mixed-breed dogs were selected for elective laparoscopic ovariectomy after obtaining written owner consent. The inclusion criteria were ASA status I and II, weight ≥10 kg, and age ≤ 10 years. Exclusion criteria were obesity, overt respiratory or cardiovascular pathologies, relevant bloodwork abnormalities, pregnancy, or major abdominal pathologies. Before surgery, all dogs were evaluated clinically based on physical, hematological, and biochemical examinations. Dogs were solid fasted for 12 h, and water was available up to 2 h before surgery.

### Anesthetic and surgical procedure

All subjects were premedicated with 0.3 mg/kg of methadone (Semfortan; Dechra, Italy; 10 mg/mL) intramuscularly (IM), and after 15 min, a cephalic vein was catheterized for the intravenous (IV) administration of drugs and fluids. Perioperative Ringer's lactate solution (Fresenius Kabi) was administered at a rate of 5 mL/kg/h IV using a specific infusion pump (IP-7700; AMP, Seoul, Korea). After 3 min of flow-by preoxygenation (8–10 L/min), anesthesia was induced with 5 mg/kg of propofol IV (Propofol; Fresenius Kabi, Isola della Scala, Italy), and thereafter, dogs were connected to the anesthetic machine (Datex Ohmeda Excel 210) using a rebreathing circuit. In all cases, isoflurane was used to maintain general anesthesia (EtIso = 1.2–1.3%), and IPPV, using volume-controlled mode (Servo-i; Maquet, Rastatt, Germany), was delivered. The Servo-i ventilator was connected to the driving gas port of the pneumatic circuit of the anesthetic machine, to use the advanced controls of the ICU ventilators to drive the bellow of the circuit, for the purposed of the study. All the spirometry data were regulated and recorded at the level of the tracheal tube of the patient using an external monitor. A schematic representation of the setting is provided as additional online supplement. The baseline ventilatory setting was standardized for all subjects and consisted of a tidal volume (VT) of 15 mL/kg, inspiratory to expiratory ratio (I:E) of 1:2, FiO_2_ >0.8, inspiratory pause of 25% of inspiratory time, and PEEP of 0 cmH_2_O. The respiratory rate (RR; breath/minute) was modified to maintain an end-tidal carbon dioxide (EtCO_2_) between 45 and 55 mmHg. Dogs were positioned in dorsal recumbency, and the metatarsal artery was catheterized to obtain main hemodynamic data (PRAM, Most Care^®^, Vytech, Padua, Italy) and to collect the arterial blood samples. During the procedure, the main respiratory parameters were recorded using a multiparametric monitor (Datex Ohmeda S/5 Anesthesia Monitor, Ohmeda, Soma Technology, Bloomfield, CT, USA). Respiratory monitoring included RR (breaths/minute), TV (mL), minute volume (MV; mL/kg/minute), EtCO_2_ (mmHg), FiO_2_ (%), peak (Ppeak; cmH_2_O), and plateau (Pplat; cmH_2_O) airway pressures, and static compliance (C_stat_, mL/cmH_2_O/kg). Cardiovascular monitoring included heart rate (HR; beats/minute), direct systolic (SAP; mmHg), diastolic (DAP; mmHg) and mean arterial pressure (MAP; mmHg), stroke volume (SV; mL), cardiac output (CO; L/min), and pulse pressure variation (PPV; %). During the procedure, if the SAP <80–90 mmHg, MAP <60–70 mmHg, and DAP <40 mmHg, an IV crystalloid bolus (5–10 ml/kg) was administered in 15 min based on the severity of hypotension and the specific characteristics of the case. If hypotension persisted, dopamine was given (10 mcg/kg/min IV). The peripheral capillary oxygen hemoglobin saturation (SpO_2_; %) was recorded using the Masimo Set Pulse CO-Oximeter^®^ Radical-7 Pulse Oximeter (Masimo Corporation, Ivine, CA, USA). End-tidal concentration of isoflurane (EtIso, %) and temperature (T®C) were also recorded.

After connection to ventilator, an initial dose of 0.4 mg/kg rocuronium was administered intravenously (Rocuronio Kabi, 10 mg/mL, Fresenius Kabi Italia S.r.l.). Neuromuscular function was evaluated by placing stimulating electrodes on the peroneal nerve and monitoring the train of four tests (TOF-Watch; Organon, Ireland). The PP was created insufflating CO_2_ in the abdominal cavity using a Veress needle with a CO_2_ insufflator (Endoflator; Karl Storz, Germany) until reaching an IAP of 8–10 mmHg. At the end of surgery, PP was gradually reduced, and the abdominal wall was sutured. Timing of surgery (skin incision, induction and discontinuation of PP, and last skin suture) was recorded in all subjects. During the recovery time, a TOF score ≥0.9 (34) was considered adequate for the extubation considering always an SpO2 ≥ 95% and continuing to monitor main physiological parameters (34). If the TOF activity was <0.9, 0.02 mg/kg of neostigmine (Intrastigmine; Torrino Medica, Italy) was administered in combination with 0.02 mg/kg of atropine (Atropina Solfato; Monico S.p.A., Italy) EV (34). Heating and oxygen support (face mask) and fluid therapy were provided until the dogs were fully awake with a rectal temperature above 37.5 °C, MAP ≥70 mmHg, and a SpO_2_ at room air (FiO_2_ 0.21) ≥95 %. Additional analgesia with 10 μg/kg of buprenorphine (Buprenodale; Dechra, Italy; 0.3 mg/mL) was administered IM based on the clinical condition of the dogs, assessed by the anesthetist in charge.

### Study protocol

Ten min after induction of PP, dogs were randomly (simple random allocation sequence generated with the chit method) divided into two groups (35): the ARMi group and the ARMc group. In the ARMi group, a sustained inflation ARM was performed. The ventilator was set to the CPAP mode, where a pressure of 40 cmH_2_O was set and maintained for 20 s (24). At the end of the maneuver, the baseline ventilatory settings were restored, adding 5 cmH_2_O of PEEP and keeping it for the rest of the surgical procedure (31). In the ARMc group, a stepwise ARM was performed. The protocol for performing the ARMc was as follows: (1) Baseline: The initial ventilatory settings included RR 6 breaths/min, I:E ratio 1:1, TV 15 mL/kg, and PEEP 0 cmH_2_O. (2) Incremental phase: PEEP was applied in steps of 5, 10, 15, and 20 cmH_2_O every five breaths, until a P_plat_ of 40 cmH_2_O was reached. This last step was maintained for 6 breaths (60 s). (3) Decremental phase and titration of best PEEP: PEEP was stepwise reduced by 2 cmH_2_O every minute, until returning to the initial baseline setting (36). This phase included the identification of the optimal level of PEEP needed to prevent alveolar collapse (considered best PEEP). At each step, C_stat_, P_peak_, and P_plat_ were monitored (Datex Ohmeda S/5 Anesthesia Monitor, Ohmeda, Soma Technology, Bloomfield, CT, USA). The best PEEP was identified as the minimum PEEP level resulting in the highest C_stat_. (4) Second incremental phase: an incremental phase was repeated as previously described, and thereafter, the baseline ventilatory settings were resumed, setting PEEP to the value of best PEEP (22, 36).

Gas exchange, respiratory system mechanics, and hemodynamic status were evaluated in four time points during the procedure: BASE (at baseline, before the pneumoperitoneum induction), PP (10 min after the creation of the PP), ARM (10 min after the recruitment), and PostPP (10 min after pneumoperitoneum resolution).

#### Respiratory system mechanics

In each dog, gas flow was measured with a calibrated heated pneumotachograph (Fleisch 125 No. 2; Fleisch, Switzerland) connected to a calibrated differential pressure transducer (Diff-Cap; Special Instruments GmbH, Germany) placed between the endotracheal tube and the Y-piece of the rebreathing circuit. Volume was obtained by numerical integration of the flow signal (37). Values of P_peak_ and P_plat_ were measured proximally to the endotracheal tube with the pressure transducer. For further data analysis, the values of the above variables were displayed and collected on a personal computer through a data conversion card. The C_stat_ of the respiratory system was calculated and indexed based on body weight as follows:


$$\begin{array}{l} {\text{C}}_{\text{statInd}}({\text{mL/cmH}}_{2}\text{O/kg})\text{ = }(\text{TV/}[{\text{P}}_{\text{plat}}-\text{PEEP}])/\text{body weight} \end{array}$$

Driving pressure (DP) was calculated as follows:


$$\begin{array}{l} \text{DP}({\text{cmH}}_{2}\text{O})={\text{P}}_{\text{plat}}-\text{PEEP} \end{array}$$

#### Gas exchange assessment

An arterial blood sample (1 mL) was collected at each time of the study, and it was immediately analyzed. The pH, PaO_2_, and PaCO_2_ were measured and corrected based on the specific body temperature at the time of sample collection. The SaO_2_ was calculated by the analyzer. The PaO_2_:FiO_2_ ratio was calculated as a descriptive index of pulmonary arterial oxygenation. In addition, the Fshunt index was calculated as described by Araos et al. (38):


$$\begin{array}{l} \text{Fshunt}(\%)=(\text{C}{\text{c}}^{\prime }{\text{O}}_{2}\;-\;{\text{CaO}}_{2})/(\text{C}{\text{c}}^{\prime }{\text{O}}_{2}\;-\;{\text{CaO}}_{2}\\ \;+3.5\text{mL}/\text{dL})\times 100 \end{array}$$

The difference between the PaCO_2_ and the EtCO_2_ (Pa-EtCO_2_; mmHg) was also calculated (39).

#### Hemodynamic evaluation

In all dogs, a metatarsal artery was catheterized and connected to the PRAM monitor using a Baxter Truwave PX-600F transducer (Baxter-Edwards, Irvine, CA, USA) and an extension line filled with saline solution. The transducer was leveled at the level of the right atrial and then zeroed. The accuracy of the signal was verified by a square wave test before starting the data collection (40). The cardiovascular parameters included in the monitoring have been SAP, MAP, DAP, SV, and CO (41, 42). The data obtained were recorded and stored automatically every 3 s on a personal computer. Data related to SV and CO were indexed by the body surface area (BSA, m^2^) of the dogs. BSA was calculated following a specific body weight (BW) to body surface (BSA) conversion table (British Small Animal Veterinary Association, Website^©^ 2021BSAVA^TM^, UK).

### Data analysis

Accepting an alpha risk of 0.05 and a beta risk of 0.2 in a one-sided test, 10 subjects were necessary in first group and 10 in the second to recognize as statistically significant a difference greater than or equal to 0.5 units for the respiratory system static compliance. The common standard deviation is assumed to be 0.43. It has been anticipated a drop-out rate of 5%. Data were analyzed using the MedCalc 14.0 software (MedCalc, Mariakerke, Belgium). Normal distribution was tested using the Shapiro–Wilk test. All data were normally distributed and therefore were reported as mean ± standard deviation (SD). Statistical analysis was performed using the two-way ANOVA test (time x treatment) for repeated measurements. If significant, Tukey's test was applied for *post-hoc* comparison between the data obtained within the same group and between the two groups. A value of *p* < 0.05 was considered statistically significant.

## Results

A total of 28 dogs were enrolled in the study; however, eight subjects were excluded because they met the exclusion criteria (*n* = 3 obesity; *n* = 2 respiratory diseases; *n* = 1 age; *n* = 2 cardiac murmur). The procedure was completed without complications in 20 dogs. There were no differences between the two groups in body weight (ARMc = 20.8 ± 4.7 kg; ARMi = 21.3 ± 7.2 kg), age (ARMc = 3.7 ± 1.1 years; ARMi = 3.5 ± 1.2 years), duration of PP (ARMc = 49.3 ± 6.3 min; ARMi = 46.8 ± 8.3 min), and surgery (ARMc = 56.8 ± 5.3 min; ARMi = 55.4 ± 9.2 min).

### Respiratory system mechanics

**Figure 1:**
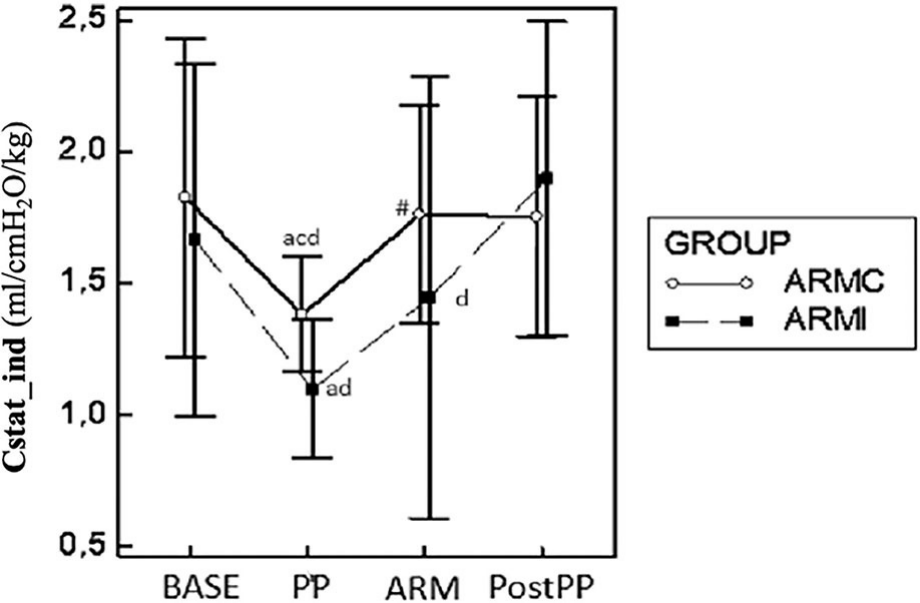
Graphical representation of the mean ± standard deviation values of C_statInd_ in 20 dogs mechanically ventilated, undergoing laparoscopic surgery. Ten dogs (ARMc) received a “stepwise” ARM, and the other (ARMi) received a “sustained inflation” ARM followed by 5 cmH_2_O of PEEP. The parameters were evaluated 10 min before (BASE) and after the induction of PP (PP), 10 min after the maneuvers (ARM), and 10 min after the discontinuation of PP (Post-PP). #*p* < 0.05 between the two groups at the same study time. ^a^*p* < 0.05 in the same group compared with BASE; ^b^*p* < 0.05 compared with PP; ^c^*p* < 0.05 compared with ARM; ^d^*p* < 0.05 compared with Post-PP.

The main respiratory parameters are described in Table 1. No differences were found in RR, VT, and PEEP. Indexed C_st_ (C_statInd_) was significantly reduced after the induction of PP (PP time) in both groups *(p* < 0.05). However, in ARMc group, C_statInd_ increased immediately after the recruitment maneuver, while, in ARMi group, the C_statInd_ increased more slowly than the ARMc group, reaching significantly higher values than PP time only after the discontinuation of the PP (Figure 1, Tables). The DP in both groups was significantly lower at PostPP (ARMc = 8.28 ± 1.47 cmH_2_O; ARMi = 6.5 ± 1.24 cmH_2_O) compared to PP time (ARMc =10.24 ± 1.65 cmH_2_O; ARMi = 9.21 ± 1.87 cmH_2_O). No differences were found in DP between the two groups of the study (Table 1).

**Table 1:** Mean ± SD of the respiratory parameters evaluated in 20 dogs mechanical ventilated, undergoing laparoscopic surgery.

**Parameter**	**Group**	**BASE**	**PP**	**ARM**	**Post-PP**
**RR** (breath/min)	**ARMc** **ARMi**	12.32 ± 4.51 11.41 ± 3.22	14.51 ± 4 15 ± 2.21	13 ± 1.24 12.40 ± 3.61	11.12 ± 3.22 12.11 ± 2.41
**TV/KG** (mL/kg)	**ARMc** **ARMi**	14.49 ± 4.13 13.20 ± 2.26	13.96 ± 2.29 14.73 ± 1.85	14.31 ± 5.54 13.84 ± 3.37	14.52 ± 4.26 13.93 ± 2.31
**C**_**statInd**_(mL/cmH_2_O/kg)	**ARMc** **ARMi**	1.78 ± 0.60 1.66 ± 0.66	1.35 ± 0.21^a^^c^^d^ 1.16 ± 0.26^a^^d^	1.71 ± 0.41^#^ 1.44 ± 0.84^d^	1.75 ± 0.45 1.89 ± 0.59
**P**_**peak**_(cmH_2_O)	**ARMc** **ARMi**	11.04 ± 1.44 10.41 ± 1.82	12.64 ± 1.5 11.41 ± 2.87	15.34 ± 3.44^a^^b^ 15.91 ± 3.54^a^^b^	14.42 ± 3.71^a^ 14.21 ± 1.47^a^^b^
**P**_**plat**_(cmH_2_O)	**ARMc** **ARMi**	10.71 ± 1.51 9.70 ± 1.82	11.53 ± 1.47 10.61 ± 1.87	14.82 ± 3.52^a^^b^ 14.52 ± 3.62^a^^b^	14.01 ± 3.61^a^ 13 ± 1.24^a^^b^
**PEEP** (cmH_2_O)	**ARMc** **ARMi**	0 0	0 0	5.7 ± 3.06 5.2 ± 0.6	5.72 ± 3.04 5
**DP** (cmH_2_O)	**ARMc** **ARMi**	8.74 ± 1.42 7 ± 1.82	10.24 ± 1.65 9.21 ± 1.87^a^^d^	9.12 ± 1.43 10.52 ± 3.62^a^^d^	8.28 ± 1.47^b^ 6.5 ± 1.24

Ten dogs (ARMc) received a “stepwise” ARM, and the other (ARMi) received a “sustained inflation” ARM. The parameters were evaluated 10 min before (BASE) and after (PP) the induction of PP, 10 min after the maneuvers (ARM), and 10 min after the discontinuation of PP (Post-PP).

# p < 0.05 between the two groups at the same study time.

a p < 0.05 in the same group compared with BASE;

b p < 0.05 compared with PP;

c p < 0.05 compared with ARM;

d p < 0.05 compared with Post-PP.

### Gas exchange parameters

**Figure 2:**
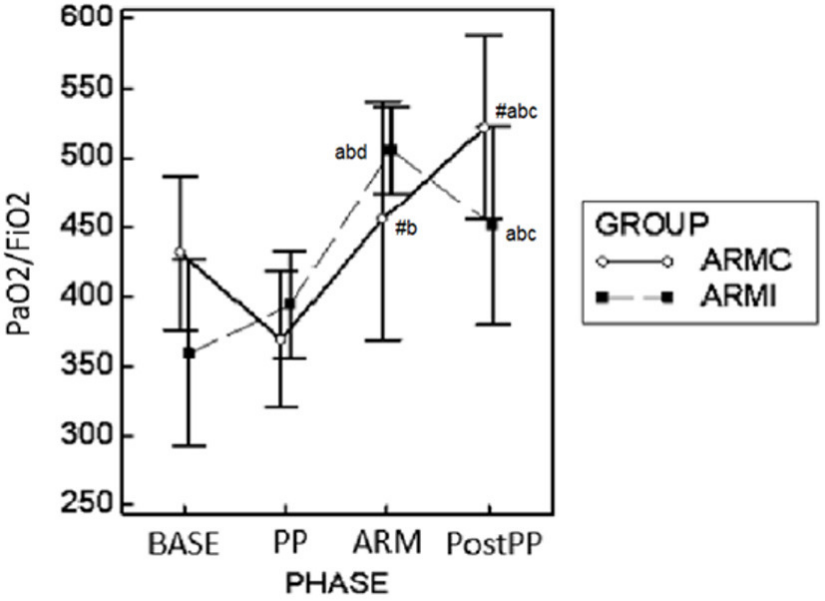
Graphical representation of the mean ± standard deviation values of PaO_2_/FiO_2_ in 20 dogs mechanically ventilated, undergoing laparoscopic surgery. Ten dogs (ARMc) received a “stepwise” ARM, and the other (ARMi) received a “sustained inflation” ARM followed by 5 cmH_2_O of PEEP. The parameters were evaluated 10 min before (BASE) and after (PP) the induction of PP, 10 min after the maneuvers (ARM), and 10 min after the discontinuation of PP (Post-PP). #*p* < 0.05 between the two groups at the same study time. ^a^*p* < 0.05 in the same group compared with BASE; ^b^*p* < 0.05 compared with PP; ^c^*p* < 0.05 compared with ARM; ^d^*p* < 0.05 compared with Post-PP.

Gas exchange parameters are reported in Table 2. There were no differences in EtCO_2_ and PaCO_2_ between the two groups. In both groups, both variables were significantly higher at PP and ARM time compared to BASE and PostPP. The Pa-EtCO_2_, in both groups, was increased significantly at PP compared to the other phases. Instead, at ARM and PostPP, it was significantly lower compared to PP time (Table 2). In both groups, PaO_2_/FiO_2_ at PP time was similar to BASE. At ARM and PostPP, it was significantly higher than BASE and PP (Figure 2). The Fshunt showed differences between the two groups at time PP and ARM. In ARMc group, it was increased significantly at PP time compared to BASE, but at PostPP, it was lower than the other phases of the study. In ARMi group, Fshunt at PP was similar to BASE. Instead, at PostPP, it has reached significantly lower values than BASE and PP (Table 2).

**Table 2:** Mean ± SD of the gas exchange parameters evaluated in 20 dogs mechanically ventilated, undergoing laparoscopic surgery.

**Parameter**	**Group**	**BASE**	**PP**	**ARM**	**Post-PP**
**pH**	**ARMc** **ARMi**	7.31 ± 0.05 7.26 ± 0.03	7.21 ± 0.02 7.18 ± 0.05	7.22 ± 0.06 7.19 ± 0.05	7.26 ± 0.11 7.21 ± 0.06
**EtCO_**2**_ (mmHg)**	**ARMc** **ARMi**	38.71 ± 2.92 40 ± 4.21	45 ± 5.60^a^^d^ 46 ± 8.81^a^^d^	46.42 ± 7.51^a^^d^ 48 ± 5.50^a^^d^	42.42 ± 8.91 45 ± 9.51
**PaCO_**2**_ (mmHg)**	**ARMc** **ARMi**	45.22 ± 6.67 45.81 ± 5.49	53.33 ± 3.74^a^^d^ 55.31 ± 10.96^a^^d^	50.31 ± 9.44^a^^d^ 54.11 ± 8.74^a^^d^	46.83 ± 15.52 51.14 ± 7.78
**PaO_**2**_/FiO_**2**_ (mmHg)**	**ARMc** **ARMi**	431.22 ± 55.11 359.81 ± 67.22	369.53 ± 49.31 394.32 ± 37.72	455.11 ± 85.90^b^^#^ 505.40 ± 31.70^a^^b^^d^	521.30 ± 66.20^#^^abc^ 450.90 ± 70.60^a^^b^^c^
**Pa-EtCO_**2**_ (mmHg)**	**ARMc** **ARMi**	7.51 ± 3.51# 5.62 ± 2.12	8.31 ± 4,44^#^^a^ 9.81 ± 4.41^a^	4.01 ± 2.61^#^^abc^ 6.21 ± 4.22^a^^b^	4.34 ± 2.21^#^^ab^ 6.11 ± 4.12^a^^b^
**SaO_**2**_ (%)**	**ARMc** **ARMi**	99 ± 0.57 99 ± 0.31	97.81 ± 1.06 98.72	97.71 ± 1.49 99 ± 0.42	99 ± 1.15 100
**Fshunt (%)**	**ARMc** **ARMi**	7.71 ± 2.82 7.92 ± 2.40	12.12 ± 3.96^#^^a^^d^ 6.61 ± 2.84	9.42 ± 5.65^#^^d^ 5.31 ± 2.22^a^	5.11 ± 3.79 4.82 ± 3.70^a^^b^

Ten dogs (ARMc) received a “stepwise” ARM, and the other (ARMi) received a “sustained inflation” ARM. The parameters were evaluated 10 min before (BASE) and after (PP) the induction of PP, 10 min after the maneuvers (ARM), and 10 min after the discontinuation of PP (PostPP).

# p < 0.05 between the two groups at the same study time.

ap < 0.05 in the same group compared with BASE; bp < 0.05 compared with PP; cp < 0.05 compared with ARM; dp < 0.05 compared with Post-PP.

### Hemodynamic parameters

Hemodynamic data at each study time are reported in Table 3. Heart rate showed no differences between the two groups. In ARMc group, HR was higher at PP (89 ± 20.53 b/min) and ARM (86 ± 15.97 b/min) compared to BASE (70 ± 14.25 b/min). The MAP showed no differences between the two groups but in both, at PP (ARMc = 113.81± 23.6 mmHg; ARMi = 105.52 ± 9.65 mmHg) and ARM (ARMc =91.14 ± 16.25 mmHg; ARMi =92.77 ± 17.56 mmHg), it was higher than BASE (ARMc = 65.85 ± 3.93 mmHg; ARMi = 76.22 ± 6.10 mmHg) and PostPP (ARMc = 75.71 ± 13.84 mmHg; 70.60 ± 2.88 mmHg). Mean values of SVI were lower in the ARMi group as compared to the ARMc group at all times of the study. There were no differences in cardiac index (CI) between groups.

**Table 3:** Mean ± SD of the hemodynamic parameters evaluated in 20 dogs mechanically ventilated, undergoing laparoscopic surgery.

**Parameter**	**Group**	**BASE**	**PP**	**ARM**	**Post-PP**
**HR (b/min)**	**ARMc** **ARMi**	70 ± 14.25 81.11 ± 17.85	89.52 ± 20.53^a^ 90.6 ± 12,67	86 ± 15.97^a^ 91 ± 14.77	84.84 ± 14.22 90.41 ± 9.71
**MAP (mmHg)**	**ARMc** **ARMi**	65.85 ± 3.93 76.22 ± 6.10	113.81± 23.6^a^^d^ 105.52 ± 9.65^a^^d^	91.14 ± 16.25^a^^d^ 92.77 ± 17.56^a^^d^	75.71 ± 13.84 70.60 ± 2.88
**SVI (L/m^2^)**	**ARMc** **ARMi**	65 ± 41^#^ 40.63 ± 16.13	65.62 ± 33.14^#^ 47.19 ± 19.65	58 ± 24.25^#^ 45.11 ± 16.24	62.51 ± 23.47^#^ 34.96 ± 3.35
**CI (L/min/m^2^)**	**ARMc** **ARMi**	3.95 ± 2.06 3.37 ± 1.76	5.47 ± 2.19 4.19 ± 1.66	4.95 ± 1.99 4.02 ± 1.37	5.26 ± 1.94 3.99 ± 0.35

Ten dogs (ARMc) received a “stepwise” ARM, and the other (ARMi) received a “sustained inflation” ARM. The parameters were evaluated 10 min before (BASE) and after (PP) the induction of PP, 10 min after the maneuvers (ARM), and 10 min after the discontinuation of PP (Post-PP).

# p < 0.05 between the two groups at the same study time.

a p < 0.05 in the same group compared with BASE;

^b^p < 0.05 compared with PP;

^c^p < 0.05 compared with ARM;

d p < 0.05 compared with Post-PP.

## Discussion

The results of this study demonstrated the efficacy of two ARM strategies for the treatment of the respiratory changes induced by laparoscopy in dogs. Our results can be summarized as follows: both ARMs used in this study showed that an “open-lung” strategy (OLs) improves respiratory function after the induction of pneumoperitoneum. Moreover, applying PEEP at the end of the ARM can prolong the beneficial effects of the OLs until the end of the surgical procedure and the discontinuation of PP. Based on our data, both strategies showed benefits in terms of improving compliance and oxygenation.

Induction of PP usually results in airway pressure increase and a reduction in respiratory system compliance at a given TV (14, 43, 44). In this study, induction of PP resulted in significant increases in P_peak_ and P_plat_ with consequential reductions in C_stat_ in both groups. Both ARMs allowed to bring C_stat_ back to values compared with BASE. In addition, the improvement of these parameters persisted even after the discontinuation of the PP, suggesting that the benefits of both maneuvers are maintained over time.

In accordance with what was mentioned, PaO_2_: FiO_2_ and Fshunt were not directly affected by PP induction in our study. Several references have shown that PP causes an increase in atelectasis but is not necessarily related to an increase in shunt or a reduction in arterial oxygenation (8, 39, 45). This apparent paradox has not yet been explained; however, even from our data, in the ARMc group, we did not detect a direct relationship between Fshunt and oxygenation compared to the development of pulmonary atelectasis. Previously, it has been shown that with increasing IAP, the degree of pulmonary shunt may decrease and arterial oxygen tension may not be affected and, in some cases, increase (8, 47). Mechanisms postulated behind an increase in PaO_2_ from elevated IAP during PP could be hypoxic pulmonary vasoconstriction, cranial diaphragm displacement with redistribution of pulmonary blood flow, and patient volume status (8, 45). Recently, the Pa-EtCO_2_ has been identified as a more reliable index of overall V/Q mismatch than the PaO_2_:FiO_2_ ratio (37) during PP. Pa-EtCO_2_ can increase due to both an increase in PaCO_2_ and a decrease in EtCO_2_. PaCO_2_ may increase when venous blood passes through perfused but atelectatic alveoli and no blood-alveolus exchange occurs. The reduction in EtCO_2_, on the contrary, usually occurs when non-perfused or poorly perfused alveoli are ventilated (39). In this study, in both groups, Pa-EtCO_2_ increased 10 min after PP induction and subsequently decreased to similar values to baseline after ARM in both groups. Probably, after PP, the increase in PaCO_2_ was not followed by a corresponding increase in EtCO_2_ inducing a significantly higher Pa-EtCo_2_ during PP compared to BASE. This finding appears to be indicative of the development of pulmonary atelectasis (perfused but unventilated alveoli). Furthermore, the significant reduction in Pa-EtCo_2_ in the ARM and PostPP phases, associated with the significant improvement in compliance, indicates the effectiveness of the two ARMs in the “open-lung” strategy.

Oxygenation and compliance did not change after the discontinuation of the PP. This result may relate to the stabilizing effect of PEEP which prolonged the results of the ARMs up to the end of the procedure. Indeed, it has been widely shown that PEEP alone is not able to give a complete alveolar recruitment, but it has a specific stabilizing role in keeping the alveoli open at the end of an ARM (48). In human medicine, a standardized PEEP level has not been identified but, usually, the “best PEEP” is titrated or set on the basis of the ventilatory needs of each patient (20, 24, 25, 28). The minimum level of PEEP to be used during laparoscopy in dogs has not yet been established. In the previous study, Di Bella et al. showed the effectiveness of applying a fixed PEEP of 5 cmH_2_O at the end of a sustained inflation recruitment maneuver (31). The data collected in our study, specifically in ARMi group, confirmed the results obtained in the previous work. The average value of best PEEP identified in the ARMc protocol was 5.7 ± 3.06 cmH_2_O. This data suggest that, probably, in dogs undergoing laparoscopic surgery, a PEEP between 5 and 6 cmH_2_O, after the recruitment maneuver, could be enough to keep an OL condition for the rest of the surgical procedure. These results, however, should be limited to the respiratory settings and timing used in this study.

After induction of the pneumoperitoneum, in agreement with the literature, we found a significant increase in MAP in both groups, which could be explained by mechanical compression of the abdominal aorta, absorption of CO_2_ with transient hypercapnia, and activation of renin–angiotensin–aldosterone axis with the production of neurohumoral factors, such as vasopressin (11, 12, 46). For this reason, an euvolemic preoperative volume status is important to minimize any cardiovascular depression associated with the pneumoperitoneum (7, 42, 50). Compression of the inferior vena cava reduces preload and is associated with the increase in afterload, which can lead to a decrease in CO, particularly in hypovolemic patients (46, 49). Several studies have shown that dogs with normal cardiovascular function can tolerate these variations of preload and afterload during laparoscopic surgery (42).

In our study, we found no significant reductions in CO, which increased slightly after PP, conversely. Moreover, based on the evaluation of pulse pressure variation (PPV), cardiac index, and stroke volume index, all dogs in the study were hemodynamically stable prior to the start of surgery and the IAP was kept between 8 and 10 mmHg (42). Studies in animal models have shown that, when the IAP is <15 mmHg, an initial increase in venous return due to compression of the splanchnic compartment and a temporary increase in CO can be observed, reducing the hemodynamic impact (49, 51).

An ARM may cause several cardiovascular side effects in humans and pigs (52–56). Both ARMc and ARMi reduce cardiac output (CO) and arterial pressure. Hemodynamic changes consist primarily of a fall in cardiac output and left ventricular preload, along with an increase in heart rate and cardiac contractility (57). A transient reduction in CO during ARMc has been demonstrated in dogs (22). An ARMi may lead to a >40% reduction in cardiovascular performance (56). In this study, no significant hemodynamic changes were detected during both recruitment maneuvers. The authors hypothesize that the absence of significant cardiovascular alterations may be associated with the slow and gradual increase and decrease in IAP, maintained at a maximum of 10 mmHg. Furthermore, all patients were assessed as hemodynamically stable prior to the start of the procedure. As demonstrated in the previous studies, this aspect could influence patients' response to pneumoperitoneum (42). Data related to SVI show that this parameter was lower in the ARMi group at all time points of the study, despite a similar CI. We do not have a specific explanation for this condition, but we can assume that it was not related to the ARM procedure.

Comparing ARMi to ARMc, we observed in both groups an improvement of lung function after the two ARMs. At the same time, we did not detect significant hemodynamic changes. Several factors should influence the choice of the most suitable ARM (36, 58–60). Specifically analyzing the two techniques, the ARMi is easier to perform and does not require advanced equipment. On the contrary, it foresees a sudden increase in intrathoracic pressures and a greater risk of hyperinflating normalized alveolar units. Differently, the stepwise maneuver let us to “titrate” the best peep to be applied subjectively to each patient. Moreover, the slow and gradual insufflation allows us to obtain a more gentle and homogeneous distribution of the flow to all atelectasis units reducing the risk of lung damage. However, it must be considered that in the previous studies showed that even the stepwise maneuver performed in the dog, while increasing respiratory compliance, can cause a slight alveolar over-distension of the non-dependent regions of the lung (61, 62). Moreover, another disadvantage is that this maneuver requires constant assessment of the compliance of the respiratory system, and the execution mode is certainly longer and more complex.

This study has several limitations. Despite the sample size calculation, data could be further increased and confirmed using a larger and less standardized population and in different surgical procedures. All dogs in the study were healthy, and different effects could be expected in more complex cases associated with respiratory and/or cardiovascular dysfunction. In this study, the effects of the OLSs have been evaluated at 10 min after the recruitment and additional studies are required to clarify the duration of the OLSs for a longer period. Similarly, it would be also interesting to evaluate the effects on the respiratory function during the postoperative period. The use of FiO_2_s >0.8% after recruitment maneuvers may have masked their real effect on oxygenation. Still, these findings are valid for animals of this weight, because in animals below 10 kg of body weight, this may no longer be true due to the different compliance of the rib cage.

## Conclusion

In conclusion, both the sustained inflation and stepwise alveolar recruitment maneuver improve similarly the lung function in dogs undergoing laparoscopic surgery, reducing the respiratory side effects of PP. In addition, the application of a PEEP at the end of the ARM allows to keep the alveolar units open and functional until the end of the procedure. The application of one of the two maneuvers should be based on the equipment and monitoring available also considering the skills of the anesthesiologist and the impact of the procedure on the individual case management.

## Data availability statement

The raw data supporting the conclusions of this article will be made available by the authors, without undue reservation.

## Ethics statement

The animal study was reviewed and approved by Comitato Etico per gli Studi Clinici sugli animali del Dipartimento dell'Emergenza e dei Trapianti di Organi. Written informed consent was obtained from the owners for the participation of their animals in this study.

## Author contributions

CD, FS, and SG: study design. CD, FS, CV, LL, LF, MS, and AC: study execution. CD, JA, CV, FS, and MS: data analysis. CD, CV, JA, MS, SG, FS, and AC: writing of the manuscript. CD, CV, JA, LL, LF, MS, SG, FS, and AC: manuscript review. All authors contributed to the article and approved the submitted version.

## Conflict of interest

Conflict of interest

The authors declare that the research was conducted in the absence of any commercial or financial relationships that could be construed as a potential conflict of interest.

## Publisher's note

All claims expressed in this article are solely those of the authors and do not necessarily represent those of their affiliated organizations, or those of the publisher, the editors and the reviewers. Any product that may be evaluated in this article, or claim that may be made by its manufacturer, is not guaranteed or endorsed by the publisher.
