# Supplementary figures and images for: Effects of two alveolar recruitment maneuvers in an “open-lung” approach during laparoscopy in dogs
Source: Front Vet Sci. 2022 Aug 18;9:904673. doi: 10.3389/fvets.2022.904673 (PMC9435385; doi:10.3389/fvets.2022.904673)

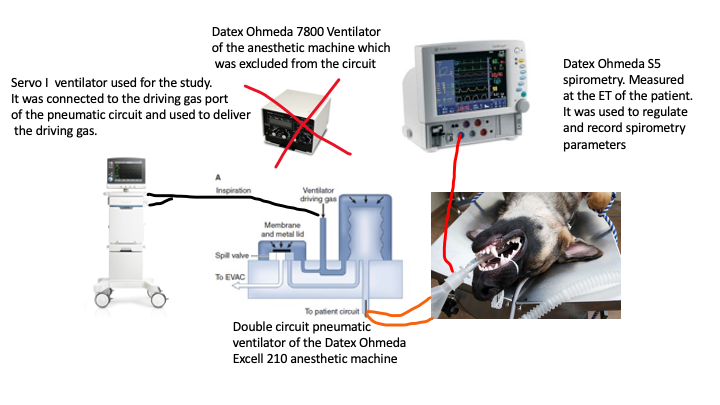

Supplement: Supplementary file 1 [file Image_1.TIFF]
